# Development of Gelatin-Coated Hydrogel Microspheres for Novel Bioink Design: A Crosslinker Study

**DOI:** 10.3390/pharmaceutics15010090

**Published:** 2022-12-27

**Authors:** Joshua Zieman, Megan Cohan, Yale Wang, Alexa De La Sancha, Muskan Kanungo, Ryan Azzouz, Rebekah Smith, Keagan Schmidt, Subha Kumpaty, Junhong Chen, Wujie Zhang

**Affiliations:** 1 BioMolecular Engineering Program, Physics and Chemistry Department, Milwaukee School of Engineering, Milwaukee, WI 53202, USA; 2 Mechanical Engineering Department, University of Wisconsin-Milwaukee, Milwaukee, WI 53211, USA; 3 Department of Biology, Midwestern State University, Wichita Falls, TX 76308, USA; 4 Mechanical Engineering Department, Milwaukee School of Engineering, Milwaukee, WI 53202, USA; 5 Pritzker School of Molecular Engineering, University of Chicago, Chicago, IL 60637, USA; 6 Chemical Sciences and Engineering Division and Center for Molecular Engineering, Argonne National Laboratory, Lemont, IL 60439, USA

**Keywords:** pectin, hydrogel, electrospray, gelatin, microspheres, bioink, vascularization

## Abstract

The development of vascularized tissue is a substantial challenge within the field of tissue engineering and regenerative medicine. Studies have shown that positively-charged microspheres exhibit dual-functions: (1) facilitation of vascularization and (2) controlled release of bioactive compounds. In this study, gelatin-coated microspheres were produced and processed with either EDC or transglutaminase, two crosslinkers. The results indicated that the processing stages did not significantly impact the size of the microspheres. EDC and transglutaminase had different effects on surface morphology and microsphere stability in a simulated colonic environment. Incorporation of EGM and TGM into bioink did not negatively impact bioprintability (as indicated by density and kinematic viscosity), and the microspheres had a uniform distribution within the scaffold. These microspheres show great potential for tissue engineering applications.

## 1. Introduction

Every day, 17 people die waiting for an organ transplant; one new person is added to the national transplant waiting list every nine minutes [1]. The demand for organs has always been present, yet the number of people placed on the waiting list continues to increase. The number of organs currently available is insufficient. Bioprinting is a branch of tissue engineering that aims to fabricate functional organs and tissues using bioink. An ideal bioink should have tunable mechanical properties and must be vascularized, biocompatible and biodegradable for successful in vivo incorporation.

A microsphere is a microscale, spherical particle typically made of polymeric materials, such as pectin and alginate. Pectin is a complex polysaccharide found within the cell walls of plants [2,3]. The structure of pectin consists of a linear chain of α-1,4-linked residues of D-galacturonic acid [4]. During pectin synthesis, methyl ester groups are added to its galacturonic acid groups. Part of these methyl ester groups are later removed for hydrogel formation. Pectin with a low degree of methylesterification gels in the presence of calcium ions, which is why it is often used as a base component in hydrogels for tissue engineering and drug delivery applications [4,5].

Positively-charged microspheres have two primary functions: (1) controlled release of bioactive molecules and (2) facilitation of vascularization of tissues [6]. In drug delivery applications, microspheres can be loaded with bioactive molecules for targeted delivery into a system [7,8]. Furthermore, in vivo studies have demonstrated that microspheres with a diameter of 200–300 µm increase blood vessel density [9]. Vascular networks are important for tissue and organ formation because they allow for blood and waste transportation. Incorporating microspheres into a bioink would promote the formation of blood vessels in the bioprinted tissue.

Gelatin, a partial hydrolysis product of collagen, is non-toxic, non-carcinogenic, biocompatible, and biodegradable [10,11,12]. Gelatin is widely used in tissue engineering [13,14,15] and drug delivery applications [16,17], particularly because studies have shown that gelatin can promote vascularization due to its arginine-glycine-aspartate (RGD) moiety [18]. However, gelatin, like pectin, has weak mechanical strength and poor resistance to hydrolysis [19]. Gelatin can be crosslinked physically, chemically, and enzymatically. Thus, catalysts such as 1-ethyl-3-(3-dimethylaminopropyl) carbodiimide hydrochloride (EDC) and enzymes like transglutaminase, can be applied to crosslink pectin and gelatin for stability improvement. Transglutaminase is a naturally occurring crosslinker that catalyzes isopeptide bond formation between glutamine and lysine amino acid residues to strengthen polymeric structures without introducing toxicity [20,21]. This enzyme improves thermal stability by increasing the number of covalent bonds. EDC forms amide bonds between the carboxylic and amine groups of molecules [22]. A benefit of using EDC is that the resulting structure is non-toxic [22].

This project aimed to develop a microsphere-based bioink to be used in the bioprinting of vascularized organ tissues. Pectin was used as the base component for the microsphere because it creates a stable hydrogel when combined with calcium cations. Pectin microspheres were produced via electrospray. The microspheres were then coated with gelatin to promote cell adhesion and vascularization. The microspheres were finally crosslinked using EDC and transglutaminase. Stability and degradation tests were performed to compare the two different crosslinking agents used. The different microspheres produced were characterized using FTIR and SEM. Bioinks containing both transglutaminase- and EDC-crosslinked spheres were prepared and used to fabricate a scaffold. The viscosity and density of each bioink was measured. The type of crosslinker could affect the performance of microsphere-based systems in drug delivery and tissue engineering applications.

## 2. Materials and Methods

### 2.1. Materials

Low methoxyl pectin (20.4% esterification) was obtained from WillPowder (Miami Beach, FL, USA). Gelatin from porcine skin (G1890), Pluronic^®^ F-127 (P2443), and pectinase were acquired from Sigma-Aldrich (St. Louis, MO, USA). 1-ethyl-3-(3-dimethylaminopropyl) carbodiimide hydrochloride (EDC, 22980), 2-morpholinoethanesulfonic acid (MES, M0606), and 10× phosphate-buffered saline (PBS, BP399) were purchased from Thermo Fisher Scientific (Waltham, MA, USA). The PBS (pH = 7.4) had a composition of 137 mM NaCl, 2.7 mM KCl, 8 mM Na_2_HPO_4_, and 2 mM KH_2_PO_4_. Transglutaminase (Moo Gloo TI) was acquired from Modernist Pantry LLC (Eliot, ME, USA). All materials were used as received.

### 2.2. Ca-Pectin Hydrogel Microsphere Fabrication

Ca-Pectin microspheres (PM) were produced via electrospray (Linari Engineering, Valpiana, Italy) using previously optimized parameters: 21 kV, 8 mm/hr, and 10 cm needle-tip-to-collector distance [23]. Briefly, a 5% (*w*/*v*) pectin solution was electrosprayed into a 150 mM CaCl_2_ solution to generate hydrogel PM through the formation of “shifted egg-box” calcium-linked junctions. Centrifugation (1200 rpm; 5 min) was used to collect PM.

### 2.3. Coating and Crosslinking of PM

**Scheme 1:**
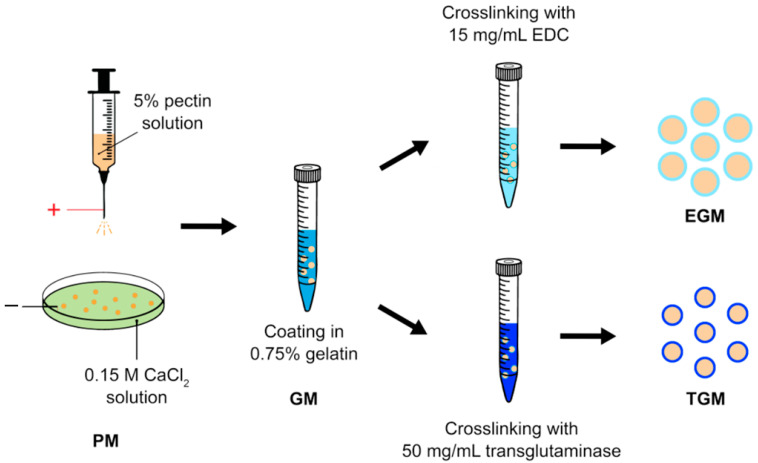
Process for producing GM, EGM and TGM samples.

PM samples were coated using previously optimized parameters: 0.75% (*w*/*v*) gelatin solution for 15 min [23]. Centrifuging (1200 rpm; 5 min) and rinsing in DI water twice, resulted in gelatin-coated pectin microspheres (GM). As illustrated in Scheme 1, GM samples were either incubated, for crosslinking, in EDC (12 h, 4 °C, 15 mg/mL; buffered with 2-morpholinoethanesulfonic acid, pH = 4.8) or transglutaminase (30 min, 20 °C, 50 mg/mL) to produce EDC-crosslinked GM (EGM) and transglutaminase-crosslinked GM (TGM), respectively.

### 2.4. Characterization Studies

In vitro analysis of morphology, size, and stability in PM, EGM, and TGM samples were investigated under an inverted microscope (EVOS XL; Thermo Fisher Scientific, Waltham, MA, USA). Samples were dispersed in PBS to analyze morphology and stability. Microsphere sizes were analyzed using NIH ImageJ software (Bethesda, MD, USA). A high-resolution cold cathode field emission scanning electron microscope (FE-SEM; Hitachi S-4800; Krefeld, Germany) was used to evaluate the surface morphology of the samples at an accelerating voltage of 5.0 kV. Samples were mounted and dried on an aluminum stub and were then coated with a layer of iridium (2 nm). An attenuated total reflection Fourier transform infrared (ATR-FTIR) spectroscopy (MIRacle 10, IR-Tracer 100; Shimadzu, Kyoto, Japan) was used to evaluate the samples structural and compositional makeup. Samples were oven-dried at 37 °C.

### 2.5. Degradability Study

In vitro enzymatic degradability of PM, TGM, and EGM were investigated to compare crosslinking agents using a 1.6% (*v*/*v*) pectinase solution to mimic a colonic environment [2]. Images were taken under an inverted microscope, in 30 s intervals for 15 min, followed by 15-min intervals until 1 h, then 1 h intervals until complete degradation [24]. Images were processed using NIH ImageJ software (Bethesda, MD) and compiled into a video. Total enzymatic degradability of each sample was conducted five times to determine the average degradation profile rates for each sample.

### 2.6. Preparation and Characterization of Microsphere-incorprated Bioink

A 5% (*w*/*v*) pectin and 20% (*w*/*v*) Pluronic^®^ F-127 solution was used as the base bioink (BI), according to our previous studies [25,26]. Electrosprayed microspheres were centrifuged to remove water. Microsphere-incorporated bioink was developed by gently mixing EGM or TGM into separate base bioink samples at a volume ratio of 1:50 (microspheres: base bioink) resulting in BIEGM and BITGM, respectively. The kinematic viscosity of the bioinks, with and without microspheres, were measured using a suspended level viscometer (Cannon Instrument Company; State College, PA). Each bioink’s density was determined by measuring the mass of 5 mL of the bioink (density = mass / volume). The viscosity measurements were conducted at 4 °C, while all the other measurements and processes were performed at room temperature (~21 °C). The bioink’s viscosity increases with an increase in temperature. The temperature of 4 °C was used in this study because the lower bioink viscosity allowed for the viscometer to be used easily [23].

### 2.7. Scaffold Fabrication Process

To fabricate a scaffold, bioink was extruded onto a Petri dish at 37 °C (AmScope TCS-100 Microscope Temperature Control Stage Slide Warmer; Irvine, CA, USA) through a 24-gauge blunt needle tip using a syringe (in a layer-by-layer pattern). After three layers were extruded to establish the foundation of the scaffold (due to the gelation of Pluronic^®^ F-127 at temperatures greater than 28 °C), warm CaCl_2_ (~37 °C, 150 mM) was added around the base of the scaffold to crosslink the pectin, chemically gelling the structure. More layers were then added. CaCl_2_ was added as the scaffold height increased. The final geometry was 2.0 cm × 2.0 cm × 0.3 cm (length × width × height). The scaffold and distribution of microspheres within it were observed under an inverted microscope [26].

## 3. Results and Discussion

### 3.1. Effects of Crosslinking on Microsphere’s Stability and Size

**Figure 1:**
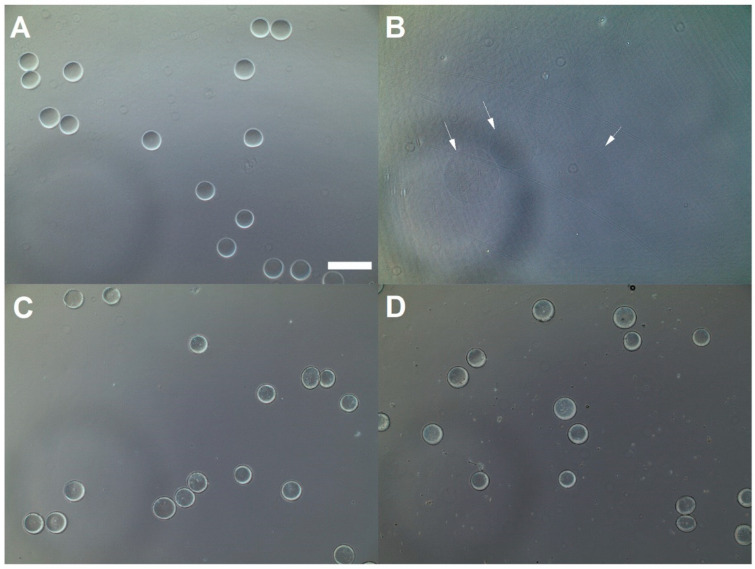
Microscopic images of microspheres: (**A**) PM in DI water; (**B**) PM in PBS; (**C**) EGM (0.75% gelatin) in PBS; (**D**) TGM (0.75% gelatin) in PBS. Arrows indicate highly swollen and disappearing PM. The scale bar represents 500 µm.

All three types of microspheres (PM, EGM, and TGM) were dispersed in PBS to examine their morphologies and stabilities (Figure 1). PM were unstable and rapidly disintegrated due to their chemical instability, especially in solutions consisting of monovalent cations and phosphate [27,28]. Specifically, phosphate (a chelating agent) can form complexes with Ca^2+^, disrupting the shifted egg-box structures stabilizing the pectin chains within the microspheres [29]. Both EGM and TGM remained stable and intact (Figure 1). Compared to PM, the improved stability of EGM and TGM is credited to the gelatin coating and crosslinking of pectin and gelatin on the microsphere surface.

**Figure 2:**
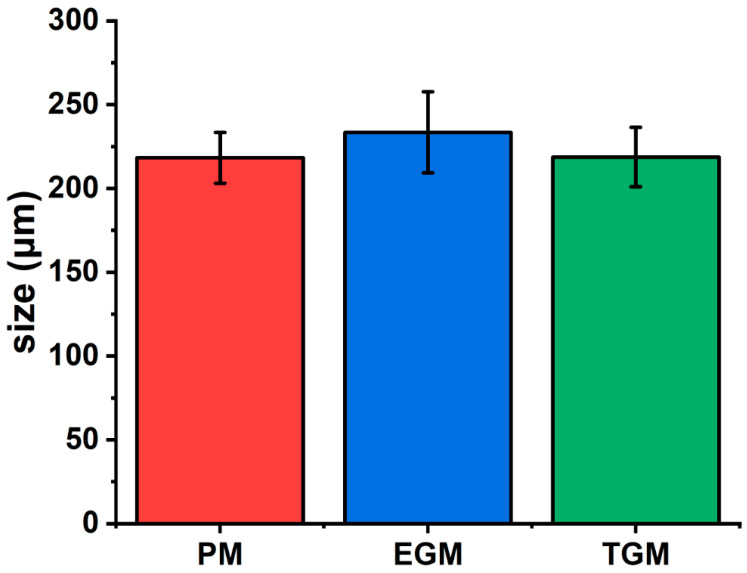
Size of different types of microspheres in DI water.

As shown in Figure 2, the sizes of EGM and TGM in PBS were similar to that of PM in water. Compared to PM, there was an 6.98% size increase for EGM and an 0.21% size increase for TGM upon gelatin-coating and crosslinking. The slight increase in size of EGM, compared to PM, can be explained by the gelatin coating and crosslinking between pectin and gelatin (interrupting and loosening Ca^2+^-pectin interactions). The relatively smaller size of TGM is likely due to the more condensed gelatin layer caused by crosslinking.

**Scheme 2:**
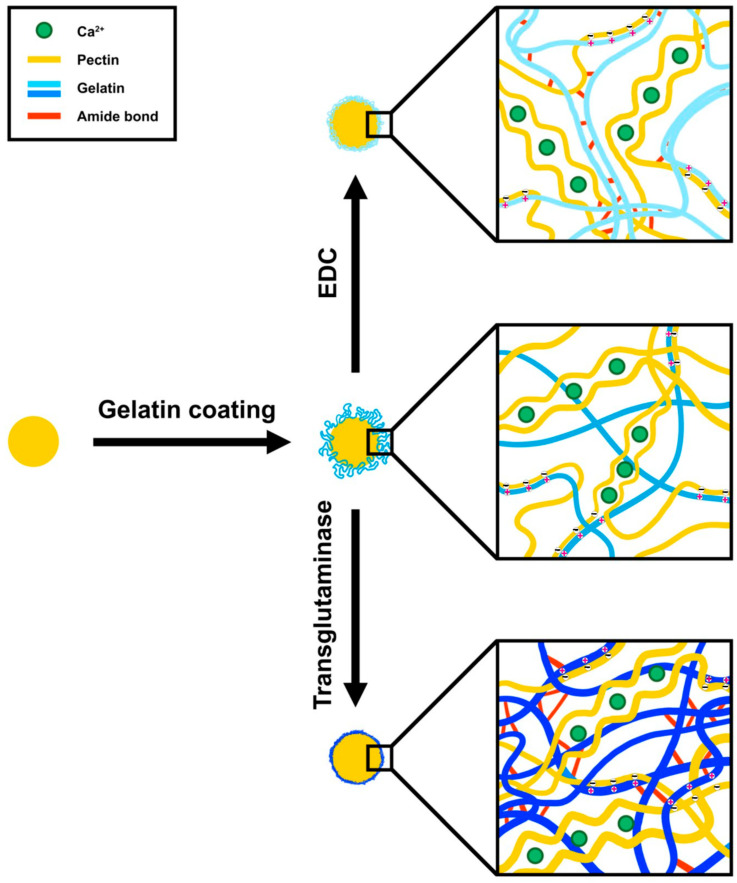
Structural changes during each step in the microsphere production process.

Gelatin contains an abundance of positively charged residues, which can be utilized to form polyelectrolyte complexes that stabilize the structure of a microsphere [23]. The catalysis mechanism of EDC causes pectin to interact with gelatin at the microspheres surface, resulting in amide bonds formation. On the other hand, crosslinking via transglutaminase results in gelatin-gelatin interactions, leading to a condensed gelatin coating (Scheme 2). The catalysis mechanism differences contributed to the slight size differences (Figure 2). Studies regarding the functionalization of pectin have also shown that RGD-pectin microspheres are larger than unmodified pectin microspheres [28].

### 3.2. Surface Morphology of Microspheres

**Figure 3:**
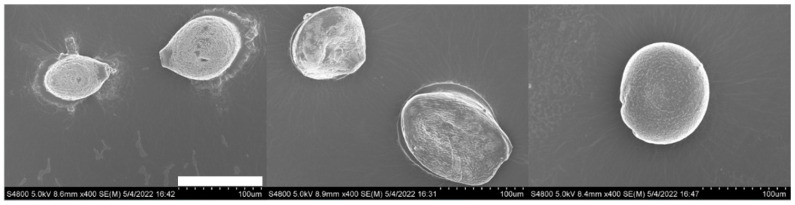
FE-SEM images of PM, EGM, and TGM, from left-right. The scale bar represents 100 µm.

FE-SEM images (Figure 3) depict the surface morphology differences between the three sample types. The white deposits present on the surface of PM are likely the result of calcium deposits [30,31]. Comparing the microspheres, PM appeared to be less round than EGM most likely due to the loss of integrity during sample preparation (also reflecting the low stability). Use of the crosslinkers, EDC or transglutaminase, seems to increase the surface smoothness and roundness (more spherical) of the microspheres. There appears to be some white deposits on the EGM surface but not TGM which could be explained by the EDC crosslinking affecting the Ca-pectin’s shifted egg-box structure. Interestingly, the transglutaminase catalysis mechanism appears to result in the smoothest surface. Many studies have shown that epithelial cell growth is improved on smooth surfaces. On the contrary, rough surfaces may aid in the healing of soft tissue [32]. Therefore, both EGM and TGM may have separate, unique tissue engineering applications, such as enhanced epithelial attachment.

### 3.3. Chemistry of Microspheres

**Figure 4:**
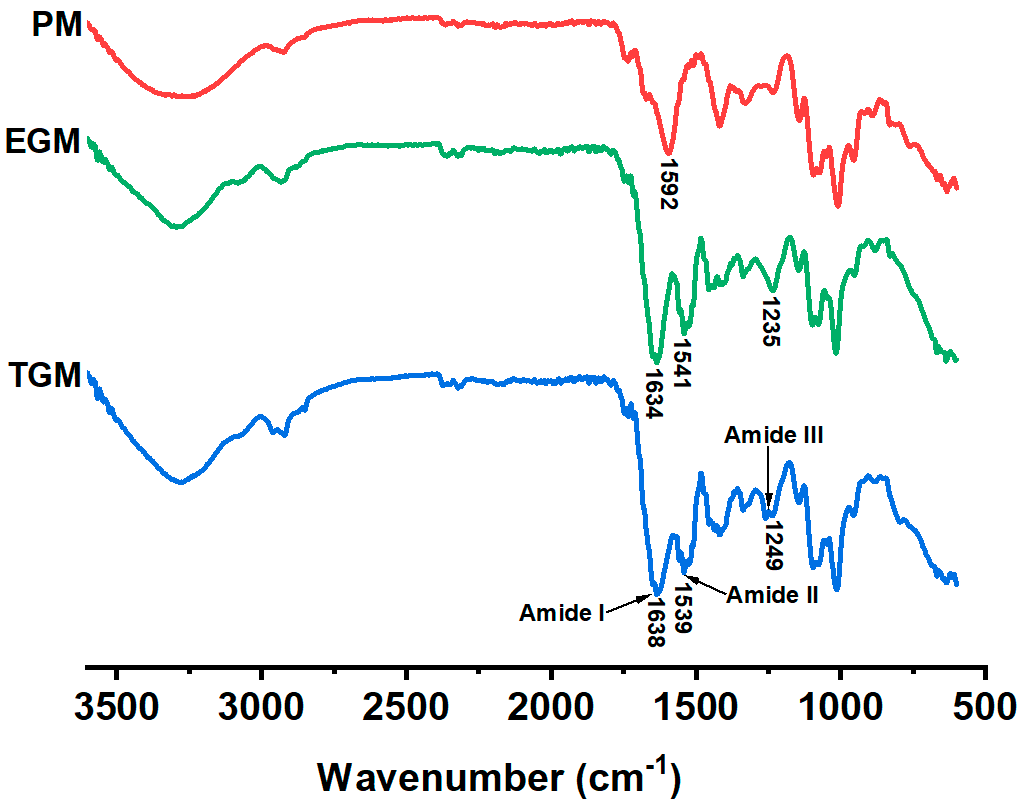
ATR-FTIR spectrum of different types of microspheres.

The surfaces of EGM and TGM contains the pectin-gelatin complexes that were formed as a result of electrostatic interaction between pectin’s carboxyl and gelatin’s amino groups during gelatin coating (Scheme 2). During EDC catalysis, it is mainly the carboxyl groups of pectin and the amino groups of gelatin that are crosslinked forming amide bonds. Transglutaminase catalyzes lysine-amide bond formation within gelatin. The FTIR-ATR spectrum of PM, EGM, and TGM is shown in Figure 4. In the PM spectrum, the band at 1592 cm^−1^ corresponds to carbonyl stretching of COO− groups [33]. Around 1634 cm^−1^ of the EGM spectrum is attributed to the COO− of pectin, amide I bond regions of gelatin, and amide bond formation [34], while the peak at 1541 cm^−1^ represents the amide II region due to the gelatin coating. The TGM spectrum shows results similar to the EGM spectrum. The lysine-amide isopeptide bond formation is confirmed by the presence of the broad bands at 1638 cm^−1^ and 1539 cm^−1^. Comparing the EGM and TGM spectra, the amide I region in EGM is more pronounced due to the contribution of the larger amount of amide bonds formed between the amine groups of the gelatin and the carboxyl groups of the pectin [35]. There is a noticeable difference in the amide III (1236 cm^−1^) between the EGM and TGM spectra.

### 3.4. Degradability of Microspheres

**Figure 5:**
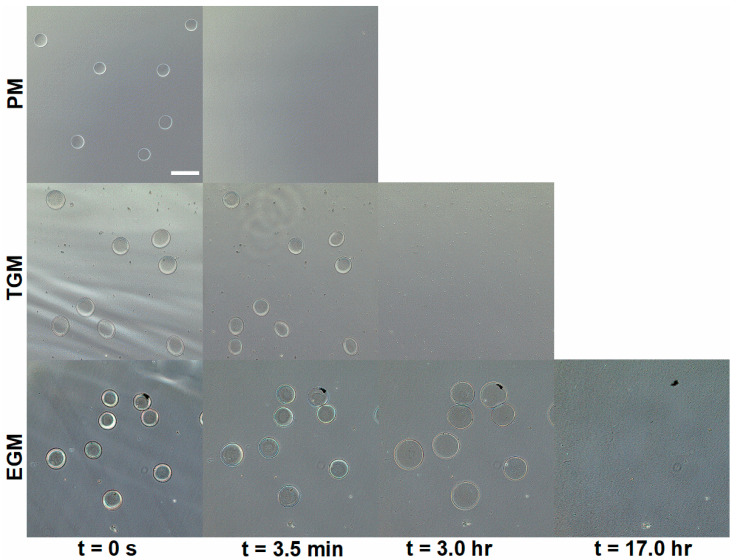
Degradation times of microspheres in 1.6% pectinase solution. The scale bar represents 400 µm.

All three types of hydrogel microspheres underwent complete enzymatic degradation within the simulated colonic fluid (Figure 5, Movies S1–S3). When PM were placed in the simulated colonic fluid, disintegration occurred immediately. The short degradation time of PM is attributed to pectinase hydrolyzing the α(1→4) glycosidic bonds in pectin to catalyze their cleavage [36]. TGM took a short time to degrade than EGM, which is in line with a previous study observing the effect of different crosslinkers on electrospun gelatin fibers [37]. EDC can catalyze amide bond formation between both pectin and gelatin, whereas transglutaminase can only catalyze amide bond formation within gelatin. Because of the crosslinking between pectin-gelatin of EGM which shields pectin from pectinase, therefore, leads to less degradable microspheres.

**Figure 6:**
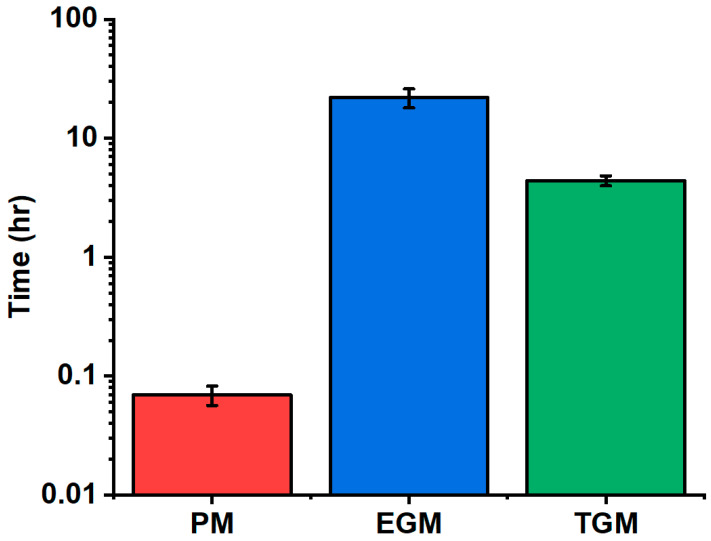
Degradation times of PM, EGM, and TGM in pectinase.

The type of sample in the simulated colonic fluid was strongly correlated to the time it took for complete degradation (Figure 6). The average degradation time of PM was 4.17 ± 0.64 min. Compared to PM, both EGM and TGM took much longer to fully degrade, at 22 ± 4 h, and 4.4 ± 0.4 h, respectively. By the time PM completely disintegrated (t = 3.5 min), EGM swelled 8.79%, while TGM shrunk 16.31% in size from their starting size (Figure 5). The swelling in EGM is likely due to the degradation starting on the inside, due to the pectin-gelatin crosslinking on the surface of the microsphere (Figure 3). The shrinking of TGM is likely because the degradation starts at the surface. Additionally, by the time TGM completely disintegrated (t = 3.0 h), EGM swelled an additional 30.24% in size from t = 3.5 min of EGM (Figure 5).

Pectin and gelatin are not crosslinked with each other in the TGM, but they are crosslinked with each other in EGM. Previous studies have suggested that the degradation ability of the microspheres depends on their structure, porosity, and degree of the inter-structure network [2]. It is likely that the type of crosslinker used slows the degradation rate of the microspheres. Additionally, the size of the microsphere may affect the time for complete disintegration in the simulated colonic fluid [38,39].

### 3.5. Effects of Microsphere-Incropration on Bioink Properties and Scaffolding

**Figure 7:**
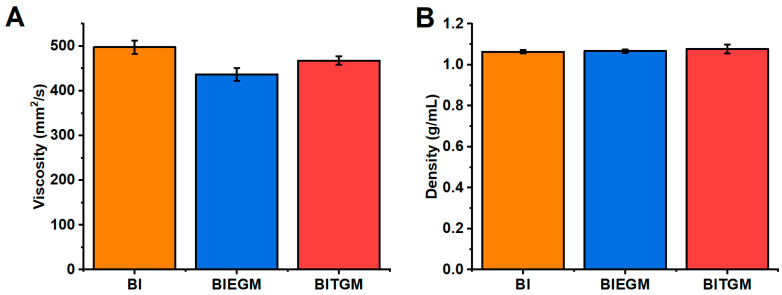
(**A**) Kinematic viscosities and (**B**) densities of BI, BIEGM, and BITGM.

The viscosity of bioink is of the determining factors of bioink’s bioprintability and scaffolding. The addition of EGM and TGM separately to base bioinks did not significantly alter the kinematic viscosity of the base bioink (Figure 7). At 4 °C, the kinematic viscosities of BI, BIEGM, and BITGM were 467.89 ± 14.35 mm^2^ s^−1^, 438.84 ± 9.27 mm^2^ s^−1^, and 439.82 ± 13.44 mm^2^ s^−1^, respectively. Comparing BIEGM and BITGM to BI, a 6.21% decrease and 5.99% decrease was determined, respectively. This may be explained by the correlation between suspension viscosity and spatial particle arrangements [40]. The densities of BI, BIEGM, and BITGM (Figure 7) were 1.0638 ± 0.0090 g/mL, 1.0773 ± 0.0218 g/mL, and 1.0671 ± 0.0082 g/mL, separately. These values indicate a 1.27% decrease for BIEGM and a 0.31% decrease for BITGM.

**Figure 8:**
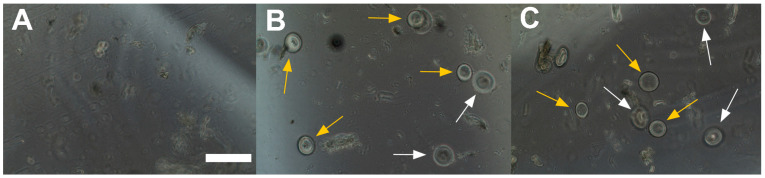
Distribution of microspheres in BIEGM (**B**) and BITGM (**C**) scaffolds compared to the bare scaffold produced using BI (**A**), showing focused (orange arrow) and unfocused (white arrow) microspheres. The scale bar represents 500 µm.

Microsphere-incorporation did not negatively affect the scaffolding process. Microspheres were intact and no physical deformities were observed. Both EGM and TGM distributed evenly in within their scaffold withing aggregation and deformation (Figure 8).

## 4. Conclusions

Gelatin-coated microspheres that undergo EDC or transglutaminase crosslinking demonstrate great potential for applications within the field of tissue engineering. The coating and catalysis steps of the process did not alter the size of microspheres considerably. Furthermore, successful gelatin coating and crosslinking by EDC and transglutaminase were confirmed by FTIR and SEM. Both EDC and transglutaminase crosslinking improved surface smoothness which is favorable for promoting vascularization. Degradability analysis under pectinase was conducted, and results showed that EGM were more stable than TGM. Upon incorporation of EGM and TGM separately into base bioink, the microspheres did not significantly change the density or kinematic viscosity, indicating that these microspheres would not negatively impact bioprintability and scaffolding. Additionally, the microspheres distributed uniformly within the bioink. In the future, encapsulation and controlled release of bioactive compounds could be investigated, along with biocompatibility characterization. Moreover, applications of this novel bioink system in tissue engineering and regenerative medicine (e.g., endometrium repair) will be investigated.

## Data Availability

Not applicable.

## Supplementary Materials

The following supporting information can be downloaded at: https://www.mdpi.com/article/10.3390/pharmaceutics15010090/s1, Movie S1: Degradation of PM; Movie S2: Degradation of EGM; Movie S3: Degradation of TGM.
