# Supplementary material for: Development of Gelatin-Coated Hydrogel Microspheres for Novel Bioink Design: A Crosslinker Study
Source: Pharmaceutics. 2022 Dec 27;15(1):90. doi: 10.3390/pharmaceutics15010090 (PMC9864922; doi:10.3390/pharmaceutics15010090)
Supplement: Supplementary file 1 [file pharmaceutics-15-00090-s001.zip › Supplementary Materials.pdf]

# Supplementary Material

**Movie S1.** Degradation of PM.

**Movie S2.** Degradation of EGM.

**Movie S3.** Degradation of TGM.

The scale bar represents 400 μm.
